# Nuclear magnetic resonance combined with genetic algorithm with linear discriminant analysis (GA-LDA) is a suitable model for discriminating urinary metabolomic profiles of individuals with glycemic disorders

**DOI:** 10.1080/07853890.2025.2566870

**Published:** 2025-10-06

**Authors:** Ângela Waleska Freire de Sousa Papa, Anne Natália Almeida de Oliveira, Rannapaula Lawrynhuk Urbano Ferreira, Geovanna Beatriz Oliveira Rosendo, Karine Cavalcanti Maurício Sena-Evangelista, Josivan Gomes Lima, Fernando Barbosa, Camilo de Lelis Medeiros de Morais, Kássio Michell Gomes de Lima, Renata Mendonça Araújo, Lucia Fatima Campos Pedrosa

**Affiliations:** ^a^ Graduate Program in Nutrition, Federal University of Rio Grande do Norte, Natal, RN, Brazil; ^b^ Nuclear Magnetic Resonance Laboratory, Institute of Chemistry, Federal University of Rio Grande do Norte, Natal, RN, Brazil; ^c^ Postgraduate Program in Health Sciences, Federal University of Rio Grande do Norte, Natal, RN, Brazil; ^d^ Nutrition Biochemistry Laboratory, Department of Nutrition, Federal University of Rio Grande do Norte, Natal, RN, Brazil; ^e^ Department of Clinical Medicine, Endocrine Unit, Federal University of Rio Grande do Norte, Natal, RN, Brazil; ^f^ ASTox Laboratory, Department of Clinical Analyses, Toxicology and Food Science, University of São Paulo - Ribeirão Preto, Ribeirão Preto, SP, Brazil; ^g^ Biological Chemistry and Chemometrics, Institute of Chemistry, Federal University of Rio Grande do Norte, Natal, RN, Brazil; ^h^ Center for Education, Science and Technology of the Inhamuns Region, State University of Ceará, Tauá, CE, Brazil

**Keywords:** Type 2 diabetes, metabolomics, urine, nuclear magnetic resonance

## Abstract

**Introduction:**

Metabolomics is essential in identifying biomarkers involved in the development and progression of diabetes. The study aimed to verify the discrimination among metabolites in the urine of individuals with glycemic alterations using Nuclear Magnetic Resonance (^1^H NMR) and multivariate analysis.

**Methods:**

The preliminary case-control study was performed with three groups: patients with type 2 diabetes (T2D, *n* = 16), patients with prediabetes (PD, *n* = 12), and control group individuals (C, *n* = 11). We obtained the 24-h urine spectra using ^1^H NMR from 39 participants. The data were analyzed using Principal Component Analysis (PCA) and supervised analyses.

**Results:**

We identified characteristic signals of various metabolites by comparing the chemical shift data with the literature. Our analysis revealed twenty-one distinct metabolic regions, emphasizing citrate, creatinine, glucose, urea, acetate, and glycine. The most pronounced differences were observed in individuals with T2D compared to groups C and PD. We evaluated a range of algorithms to determine the optimal model. The Genetic Algorithm with Linear Discriminant Analysis (GA-LDA) model exhibited remarkable accuracy, sensitivity, and specificity rates of 100% in discriminating between the studied groups.

**Conclusion:**

The ^1^H NMR and the GA-LDA model are promising methods for discriminating urine metabolomic profiles in case-control studies involving individuals with PD and T2D.

## GRAPHICAL ABSTRACT

**Figure:**
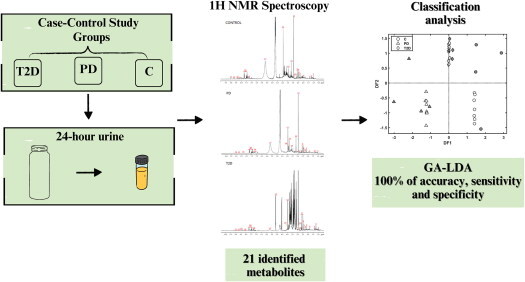

## Introduction

Hyperglycemia is a metabolic change widely used as a biomarker for screening and diagnosing diabetes. Considering the increasing prevalence of DM and its complications, studies about metabolomics and type 2 diabetes (T2D) are essential to identify the biomarkers involved in the development and progression of the disease. Furthermore, when integrated with laboratory tests, they can act as a tool to aid in diagnosing T2D [1].

Metabolomics identifies low molecular weight molecules capable of indicating alterations related to the presence of diseases. Circulating metabolites (blood) are tightly regulated, whereas excretory metabolites (urine) exhibit more significant variability, providing insights that would not be possible solely through blood analysis [2]. Although both are used in studies, urine offers an advantage because it is readily available, easily obtained and less complex than other body fluids. This distinction, along with the kidney’s ability to regulate unusually high or low metabolite concentrations, makes urine an especially valuable biofluid for medical diagnostics [3].

Playdon et al. (2016) highlight the reliability of urine as a complementary biomarker to serum in epidemiological nutrimetabolomic research, as it contains metabolites derived from habitual diets [4]. Overall, urinary metabolites are associated with multiple metabolic pathways related to cardiometabolic conditions, gut microbiota activity, energy metabolism, and dietary intake [5,6]. These metabolites result from the activity of genes and proteins, reflecting both genetic and environmental aspects and medications [7].

Urine metabolic profiles have been identified by high-resolution Nuclear Magnetic Resonance (NMR) spectroscopy, gas chromatography-mass spectrometry (GC-MS), direct flow injection tandem mass spectrometry (DFI/LC-MS/MS), inductively coupled plasma mass spectrometry (ICP-MS), and high-performance liquid chromatography (HPLC) with ultraviolet (UV) or fluorescence (FD) detection techniques. Among these, NMR is a well-established technique applied to excite magnetic properties of atomic nuclei, generating consistent results of metabolic signals present in biological matrices. Additionally, it is the method of choice for global or untargeted metabolomic analysis of urine [3].

Metabolite identification by ^1^H NMR spectroscopy relies primarily on resonance assignments from proton and bidimensional NMR spectra facilitated by spectral databases and literature references for known compounds. In metabolomics studies, chemometric tools are widely used to analyze spectral patterns, aiding in sample classification and biomarker discovery. These tools include both unsupervised and supervised multivariate analyses [8].

Principal Component Analysis (PCA), an unsupervised technique that aims to reduce the data’s dimensionality and preserve the samples’ essential characteristics [9]. In contrast, supervised analysis methods explore models capable of predicting the properties of unknown samples, providing benefits that include understanding disease mechanisms [6].

Genetic algorithm (GA) is a supervised feature selection algorithm inspired by Mendelian genetics [10], where a set of initial variables undergo processes like selection, cross-over combinations, and mutations until the fittest set of variables is found [11]. When coupled with linear discriminant analysis (GA-LDA), the fitness function that optimizes the variables selected during the processes above is a linear classifier that maximizes the class separation by minimizing the inner distance of each sample from the mean of its class while maximizing the outer distance between different classes [12]. The final classification model is then performed employing linear discrimination to the selected set of variables.

T2D triggers variations in metabolites of amino acids (isoleucine, leucine, and valine), monosaccharides (glucose and fructose), fatty acids (linoleic acid and palmitic acid), and more. These alterations are closely related to the onset of T2D [13,14] the use of metformin and glipizide [15] and patient prognosis [16]. Among these metabolites, elevated branched-chain amino acids (BCAA) analysed by liquid chromatography–mass spectrometry were related to insulin resistance and obesity in T2D patients [15]. Evidence from a systematic review also suggests that these classes of metabolites are altered in individuals with prediabetes [13].

The Study of Health in Pomerania identified several urinary metabolites associated with DM in men and women who developed the disease over five years. Glucose and lactate concentrations analysed by ^1^H NMR were positively related to DM incidents, with an increased risk of up to 1.8 times, as measured by the increase in standard deviation [17]. A previous cohort study found in patients with suspected stable angina an increased risk of T2D (19–25%) associated with choline-related metabolites such as betaine, N, N-dimethylglycine, and sarcosine measured by GC-MS/MS [18].

Concerning recent advancements in omics linked with markers of diabetes complications, the 1H NMR has continued to be a cornerstone of modern medical practice. High BCAAs (isoleucine, leucine, and valine), pseudouridine, and threonine measured by target ^1^H NMR in patients with early-stage type 1 diabetes from Finnish Diabetic Nephropathy (FinnDiane) study were associated with worse albuminuria and progression of diabetic nephropathy [19]. More recently, the DIAMOND study using multivariate analysis based on ^1^H NMR of urine metabolites identified the combination of clinical factors and urinary metabolites (alanine, choline, N-phenylacetylglycine, and trigonelline) as a highly valuable hub of biomarkers for predicting biopsy-confirmed diabetic nephropathy in T2D patients [20]. It is also remarkable to consider the benefits and diagnostic/prognostic value of low-field, near-portable benchtop NMR spectrometers designed for multicomponent metabolomics analysis of targeted and untargeted urinary biomarkers in T2D patients [21].

In line with these findings, the literature shows gaps related to the GA-LDA model applied in studies involving urine analysis by ^1^H NMR in individuals with T2D combined with a multivariate model. Thus, this study’s novelty is based on metabolomic approaches to choose urine as a biological matrix in case-control studies that aim to discriminate primary metabolites that are markers of disorders in protein, carbohydrate and lipid metabolism involved in glycemic homeostasis and diabetes pathophysiology. Thus, we conducted this study to analyze metabolites in urine samples from individuals diagnosed with prediabetes (PD) or T2D, using ^1^H NMR as a spectroscopic method and multivariate analysis.

## Methods

### Study design and population

The preliminary case-control study consisted of three groups: patients with type 2 diabetes (T2D, *n* = 16), patients with prediabetes (PD, *n* = 12), and the control group (C, *n* = 11), which was composed of individuals without glycemic alterations. Patients were recruited from Endocrinology Clinic of Onofre Lopes University Hospital (HUOL) of the Federal University of Rio Grande do Norte (UFRN), Natal, Brazil. Patients were recruited from 2019 to 2021 during clinical care with an endocrinologist and we enrolled a control group from clinical triage at the same hospital or invited them through social media. The Ethics and Research Committee of Hospital approved the study under protocol no. 3.127.214. All experiments were performed according to relevant local guidelines and regulations. The study had adhered to the principles stated in the ‘Helsinki Declaration.

The inclusion criteria for T2D were individuals of both sexes, adults and older people, diagnosed by fasting glycemia ≥126 mg/dL, blood glucose 2 h after 75 g of oral glutol >200 mg/dL, or, random blood glucose >200, or glycated hemoglobin (HbA1c) (≥6.5%) and symptoms compatible with diabetes. Patients with prediabetes (PD group) were recruited according to fasting blood glucose (≥100 *e* < 126mg/dL), or blood glucose 2 h after 75 g (≥140 *e* < 200 mg/dL), or HbA1c (≥5.7 *e* < 6.5%) [22]. Those individuals without glycemia changes were recruited as controls. Individuals who were pregnant or lactating, renal patients undergoing hemodialysis and who underwent bariatric surgery, and individuals with digestive diseases, acute infections, mental illnesses, or some memory disorders were excluded. We collected sociodemographic variables through a questionnaire applied to participants. Written informed consent was obtained from all subjects or their legal guardian(s).

### Clinical and dietary characteristics

The BMI was obtained from the participant’s weights and heights and interpreted according to the reference values of the World Health Organization [23]. The waist circumference (WC) was classified as a higher risk of metabolic alteration when > 88 cm in females and > 102 cm in males [24]. Blood pressure (BP) was measured and classified following the Brazilian Arterial Hypertension Guidelines [25]. All participantes had blood sample collected after fasting for 8–12 h. We use the Wiener (Wiener Laboratories, Rosario, Argentina) to analyse kits to determine glucose, total cholesterol (TC), triglycerides (TG), low density lipoprotein (LDL-c), and high-density lipoprotein (HDL-c) concentrations which were categorized according to guidelines [26]. Glycated hemoglobin (HbA1c) was measured by high-performance liquid chromatography using the Variant II automated analyzer. Insulin concentrations were determined by chemiluminescence, using the Immulite 2000 equipment. Insulin resistance was quantified by homeostatic model assessment-insulin resistance (HOMA–IR) defined as [fasting glucose (mmol/L) × insulin mU/L)/22.5] [27].

Data on food intake were obtained from collecting two R24h dietary recalls (R24h) with an interval of 30 to 45 days. The foods consumed were converted into nutrient values using the Virtual Nutri Plus software (version 2.0), and data processing was performed using the Multiple Source Method (https://msm.dife.de) to correct intra-individual variability and estimate of habitual food consumption [28].

### ^1^H NMR urine analysis

All overnight urine samples were collected without additives using polyethylene bottles previously sanitized. Participants were instructed to start collecting from the day’s second urine until the next day’s first urine. During 24-hour collection, the urine remained under refrigeration. For analytical proceedings, the temperature, time for storage during urine collection, and transportation to the laboratory were monitored. The urine was aliquoted, and samples were stored at −80 °C until NMR analysis. Before NMR analysis, urine samples were thawed and centrifuged at 1200 × g for 10 min.

Urine samples were analyzed using a Bruker Ascend 300 MHz NMR spectrometer (Rheinstetten, Germany) at a fixed temperature of 298 K (25 °C) to ensure spectral consistency and minimize chemical shift variations. Samples were prepared by mixing 500 µL of urine with 100 µL of deuterated water (D_2_O, 99.9 atom% D, Sigma-Aldrich) in 5 mm NMR tubes (Bruker, Germany). The spectra were acquired using the *zgpr* pulse sequence for water suppression, with a spectral width of 16 ppm, 128 scans, a relaxation delay of 2 s, and a 90° pulse width of 9.60 μs (0 dB). To ensure accurate chemical shift assignment, additional spectra were obtained without solvent suppression for metabolite identification. All data were processed in TopSpin 3.2 (Bruker, USA) for Fourier transformation, phase and baseline correction, followed by spectral preprocessing, including vector normalization and mean-centering, using MATLAB R2014b (MathWorks, USA) with PLS Toolbox 7.9.3 (Eigenvector Research, USA).

The spectra were acquired with water peak suppression in the −3,313 to 12,706 ppm range with a resolution of 0.001 ppm, generating 32,768 data points for each spectrum. Raw spectra were treated by phase correction, followed by (1) track selection between 0 and 10 ppm (20,457 data points for each spectrum) to remove chemical shifts below 0 ppm with zero intensity (above 10 ppm); (2) baseline correction to suppress baseline deviations; and (3) vector normalization to reduce physical effects that cause an offset in the spectrum, that is, systematic variations in the Y-axis of the spectrum, caused by possible temperature variations during measurements or different injections of sample quantity [29]. The vector normalization, also known as L2-normalization, scales the spectral data such that each spectrum array has a length of 1 (norm = 1) [30]. All data were mean-centered before the multivariate analysis [11].

### Data processing and multivariate analysis

Pre-processed data (baseline correction followed by vector normalization) were first analyzed by Principal Component Analysis (PCA), followed by supervised analysis, to maximize separation between the control, PD, and T2D groups. The pre-processed data were split into training (∼70%, 27 samples) and test (∼30%, 12 samples) sets using the Kennard-Stone (KS) algorithm [11,31] (Supplementary Table S1).

A series of supervised classification algorithms were tested with the dataset: Genetic Algorithm with Linear Discriminant Analysis (GA-LDA), PCA with Linear Discriminant Analysis (PCA-LDA) [32], PCA with Quadratic Discriminant Analysis (PCA-QDA) [33], Partial Least Squares with Discriminant Analysis (PLS-DA) [25], and Support Vector Machines (SVM) [34].

The GA-LDA is a variable selection technique based on the Genetic Algorithm (GA), followed by a Linear Discriminant Analysis (LDA). First, the GA-LDA is applied to the training set to reduce the spectral variables into a small group of greater importance based on an evolutionary process [10]. Then, the LDA is applied to the data set for these variables to obtain the classification [10]. We chose the variables by GA on the same intensity scale as the pre-processed data and considered the lowest risk of classification error. The GA-LDA model was built with 200 generations, each having 400 chromosomes. Probabilities of crossover and mutation were set to 60 and 1%, respectively [12]. The algorithm was repeated three times, and the model that led to the best result in terms of classification was selected. The GA-LDA model selected a total of 21 variables.

### Validation of the models

Classification models were validated by leave-one-patient-out cross-validation, in which, for each iteration of cross-validation, one patient is transferred to the validation set [10]. They were validated by external validation with a test set generated in the final validation of the models. Accuracy (AC), sensitivity (SENS), and specificity (SPEC) metrics were calculated for each set (training, cross-validation, and test), according to the following equations:

(1) $$\text{AC}(\%)=(\frac{\text{TP}+\text{TN}}{\text{TP}+\text{FP}+\text{TN}+\text{FN}})\times 100$$

(2) $$\text{SENS}(\%)=(\frac{\text{TP}}{\text{TP}+\text{FN}})\times 100$$

(3) $$\text{SPEC}(\%)=(\frac{\text{TN}}{\text{TN}+\text{FP}})\times 100$$

where TP stands for true positives; TN stands for true negatives; FP stands for false positives; and, FN stands for false negatives.

### Statistical analysis

The descriptive analysis employed either mean and standard deviation or median and interquartile range to summarize the data. Normality of variables was assessed using the Shapiro-Wilk test. Categorical variables were examined using the chi-square test. Differences between means were verified using ANOVA, followed by Tukey’s post-test. For variables with non-normal distributions, the Kruskal-Wallis test was applied, followed by Dunn’s post-test. Statistical significance was calculated for each spectral variable (chemical shift) selected by GA-LDA and defined as *p* < 0.05. A total of 39 samples (12 from the PD group, 16 from the T2D group, and 11 from the control group) were studied with a statistical power of 59% for distinguishing between controls and the PD group, 64% for distinguishing between controls and the T2D group, and 74% for distinguishing between the PD and T2D groups, based on a F-test applied to the pre-processed spectral data.

## Results

### Characteristics of the participants

This study included 39 participants: 12 from the PD group, 16 from the T2D group, and 11 from the C group. The mean ages and standard deviations were 51 (8), 51 (11), and 43 (6) years, respectively, for the C, PD, and T2D groups. Females were the most frequent in the sample, contributing to 66.6%. The differences in fasting glycemia and HbA1c reported between the groups validate the design of the case-control study, mainly regarding the criteria met in the selection of the control group (Table 1; Supplementary Table S2). Demographic and clinical characteristics across the three groups are described in Table 1. The diet composition does not differ among groups concerning adequate caloric distribution of macronutrients. (Supplementary Table S3).

**Table 1.** Demographic and clinical characteristics of the participants.

Variables	All	C	PD	T2D	*p*-value
	(*n* = 39)	(*n* = 11)	(*n* = 12)	(*n* = 16)	
Sex (%)					
Female	26 (67)	7 (63)	9 (75)	10 (62,5)	
Male	13 (33)	4 (36)	3 (25)	6 (37,5)	
Age (years)^a^	49 (10)	43 (6)	51 (8)	51 (11)	0,048
BMI (kg/m²)^a^	29 (4)	27 (4)	31 (6)	29 (3)	0,133
WC (cm)^b,c^	95 (76–121)	84 (82–88)^B^	104 (84–106)^A,B^	99 (91–105)^A^	**0,015**
Fasting glucose (mmol/L)^b,d^	122 (81–291)	88 (83–96)^B^	102 (96–105)^A,B^	150 (111–174)^A^	**0,000**
HbA1c (%)^b,d^	6 (5–12)	5 (5–6)^B^	6 (6-6)^A^	7 (6–9)^A^	**0,000**
HOMA-IR^b,d^	4,2 (0,4–20,4)	1,5 (0,5-1,9)^B^	1,9 (1,0–5,8)^A,B^	4,0 (2,0–10,6)^A^	**0,004**
TC (mg/dL)^a,d^	182 (47)	184 (60)	193 (32)	172 (47)	0,503
TG (mg/dL)^a,d^	128 (59)	102 (55)	140 (62)	137 (57)	0,234
HDL-c (mmol/L)^a,d^	47 (10)	50 (8)	49 (11)	43 (11)	0,097
LDL-c¹ (mmol/L)^a,d^	113 (34)	113,2 (34)	117,8 (27)	102,2 (38)	0,290

C = control, PD = prediabetes and T2D = type 2 diabetes groups. BMI = body mass index, WC = Waist circumference, HbA1c = glycated hemoglobin, HOMA-IR = homeostatic model assessment-insulin resistance, TC = total cholesterol, TG = Triglycerides, HDL-c = high density lipoprotein, LDL-c = low density lipoprotein. Data are presented as n (%), mean (standard deviation).

^a^ MEdian (interquartile range).

^b^ Significant values are given in bold (*p* < 0.05, ANOVA, Kruskal-Wallis, and Chi-square tests). Different capital letters in the lines indicate statistical difference using the Tukey.

^c^ or Dunn.

^d^ test (*p* < 0.05).

### ^1^H NMR spectroscopy of urine from groups of participants

**Figure 1.**
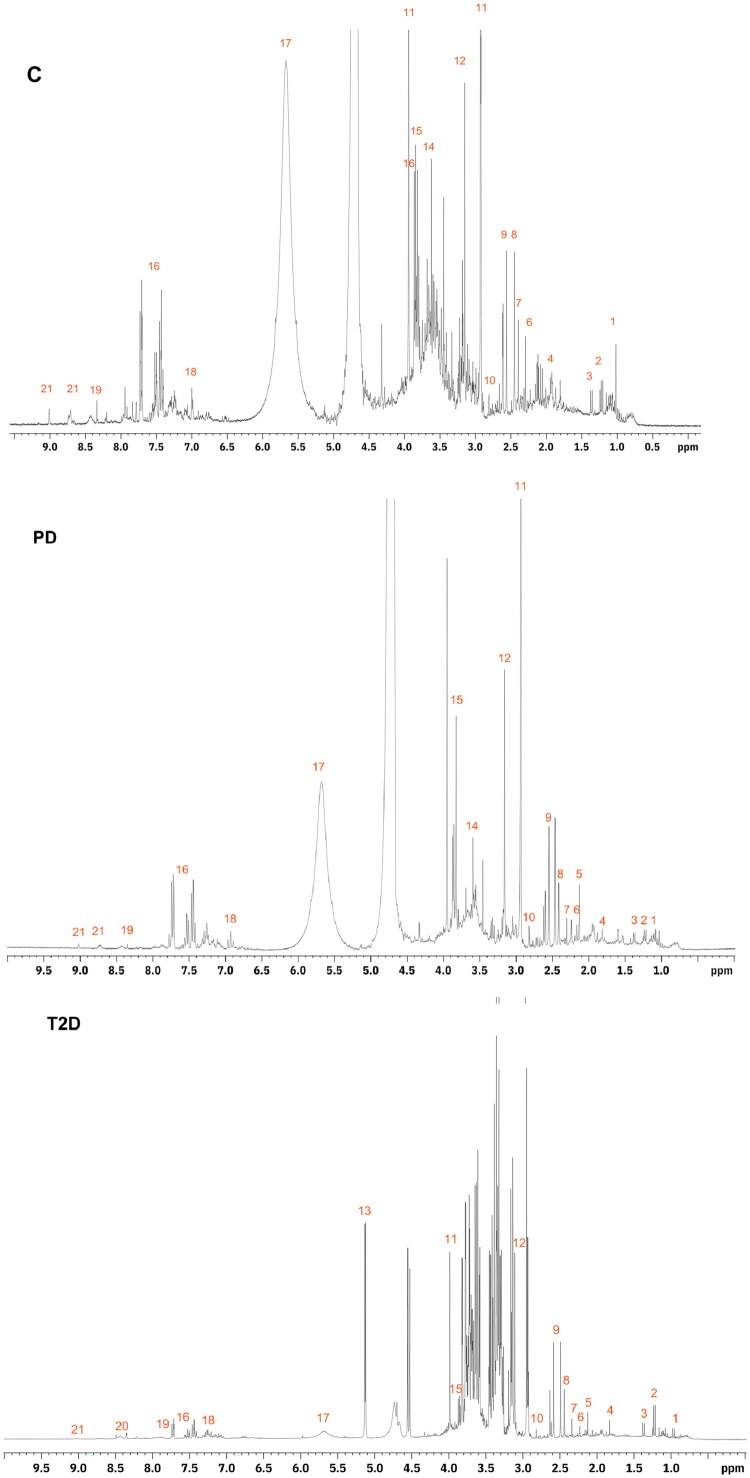
**1H** NMR spectra of urine from participants in the control (C), prediabetes (PD) and type 2 diabetes (T2D) groups. Numbers indicate the following metabolites: 1: valine; 2: OH butyrate; 3: lactate; 4 acetate; 5: acetone; 6: acetoacetate; 7: pyruvate; 8, succinate; 9: citrate; 10: creatine; 11: creatinine; 12: malonate; 13: glucose; 14: glycine; 15: glycolate; 16: hippurate; 17: urea; 18: phenylalanine; 19: hypoxanthine; 20: formate; 21: trigonelline.

The ^1^H NMR spectra of each urine sample (*n* = 39) were acquired and are shown in Figure 1. The group variance for these data was 2.09 × 10^−6^ for controls (coefficient of variation = 0.90), 1.56 × 10^−6^ for the PD group (coefficient of variation = 0.90), and 3.25 × 10^−6^ for the T2D group (coefficient of variation = 1.23). Visual differences between the spectra of the groups were observed between 5.3 and 6.1 ppm, in which the T2D group was less intense and in the 2.8 to 4.2 pp range (Figure 1). Generally, the T2D group presented greater signal intensity than the control and PD, which have similar profiles (Supplementary Figures S1, S2, S3). Table 2 describes the characteristic regions of metabolites derived from proteins, carbohydrates, lipids, and alkaloids. We identified the metabolites by comparing the chemical shifts obtained in the ^1^H NMR spectra with data from the literature. In addition, we chose studies carried out with T2D [35,36] in this step.

**Figure 2.**
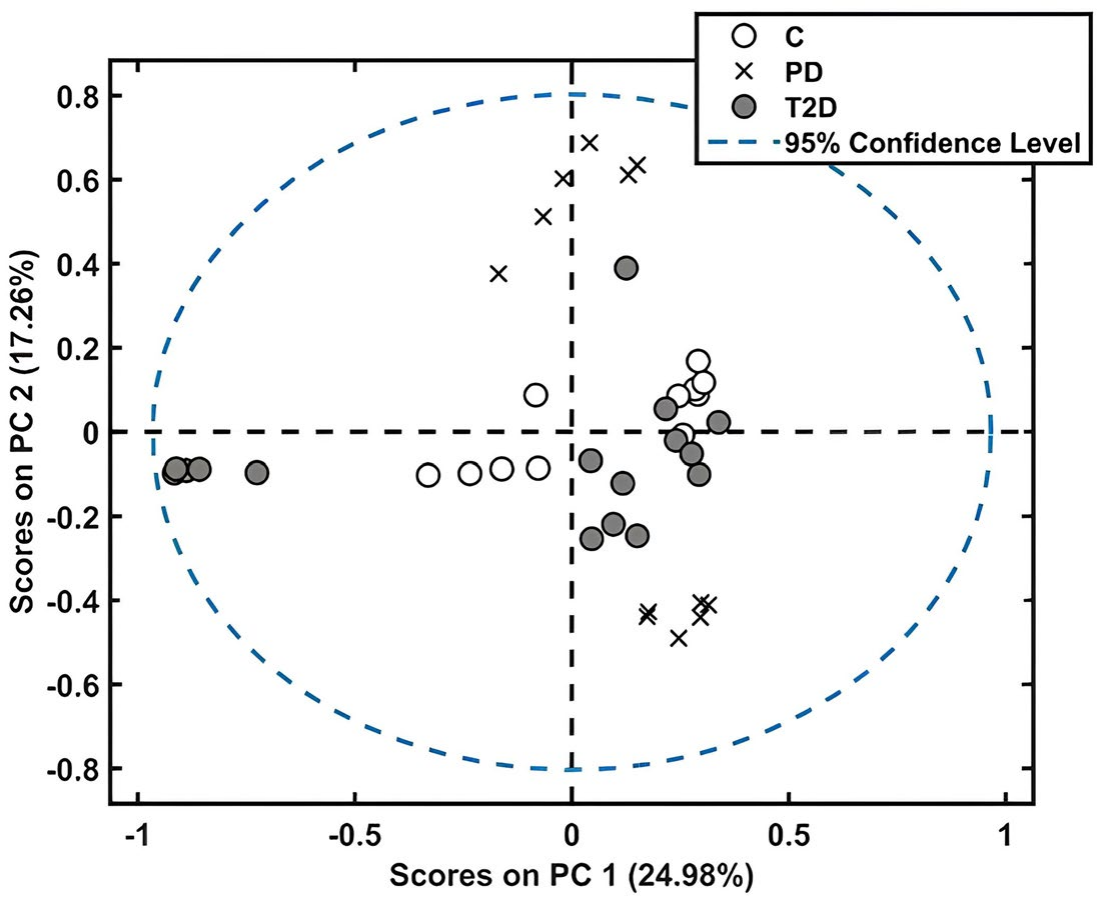
Scores for the PCA model for samples from the control (C), prediabetes (PD), and type 2 diabetes (T2D) groups. The percentage inside each parenthesis represents the variance explained in each PC.

**Figure 3.**
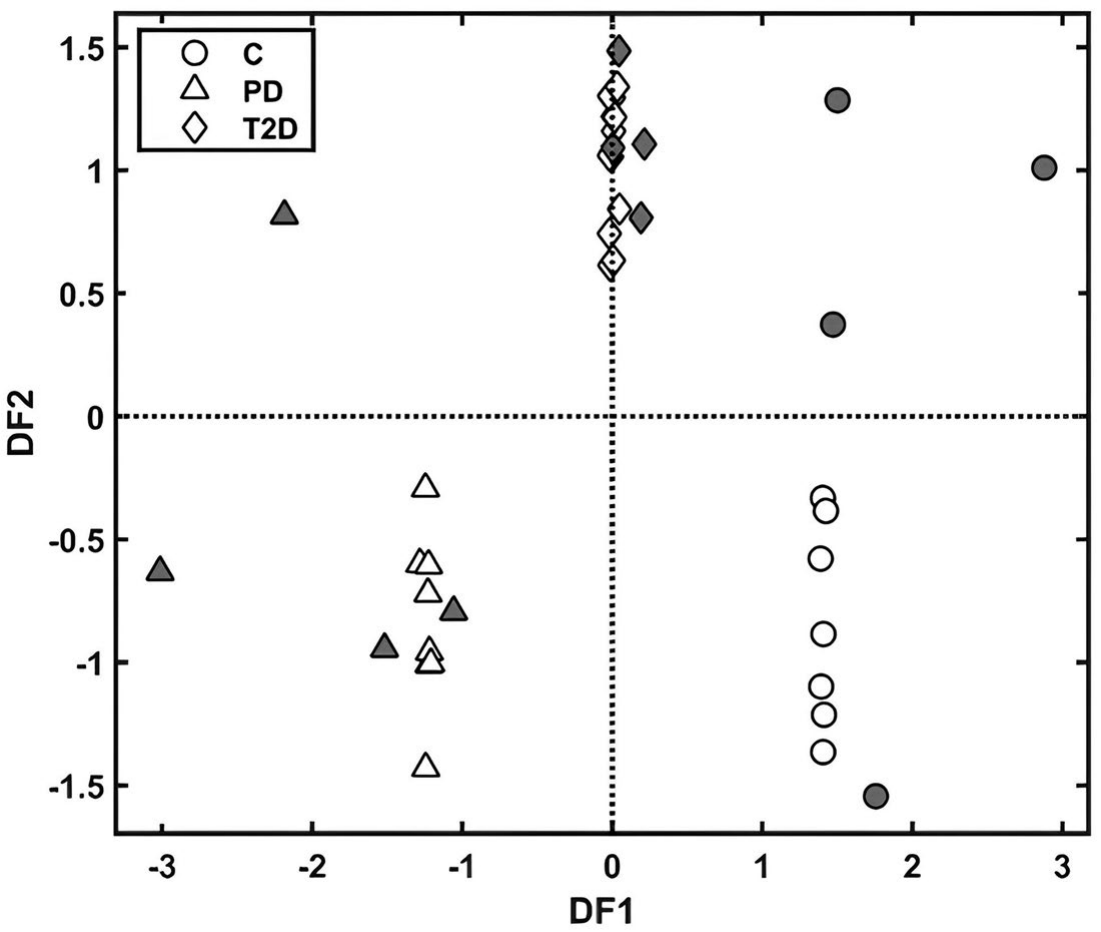
Discriminant functions 1 (DF1) and 2 (DF2) for the GA-LDA model for the control (C), prediabetes (PD) and type 2 diabetes (T2D) groups. Training (empty symbol), testing (filled symbol).

**Table 2.** Characteristic chemical shifts of metabolites identified in urine from the control (C), prediabetes (PD), and type 2 diabetes (T2D) groups.

Code	Metabolites	Chemical shift ppm (multiplicity)			Chemical groups
		C	PD	T2D	
Protein metabolism					
1	Valine	1.02 (d)	1.00 (d)	0.98 (d)	[CH3]
10	Creatine	2.92 (s)	2.82 (s)	2.92 (s)	[NCH3]
11	Creatinine	2.93 (s)	2.95 (s)	2.94 (s)	[NCH3]
14	Glycine	3.45 (s)	3.59 (s)	–	[CH2]
17	Urea	5.70 (sl)	5.70 (sl)	5.70 (sl)	[CO(NH2)]
18	Phenylalanine	7.28 (m)	7.10-7.30 (m)	7.25 (m)	[CH]
Fatty acid metabolism					
2	OH Butyrate	1.22 (d)	1.23 (d)	1.22 (d)	[CH3]
4	Acetate	1.92 (s)	1.88 (s)	1.83 (s)	[CH3]
5	Acetone	–	2.13 (s)	2.13 (s)	[CH3]
6	Acetoacetate	2.29 (s)	2.25 (s)	2.23 (s)	[CH3]
12	Malonate	3.15 (s)	3.16 (s)	3.11 (s)	[CH2]
Carbohydrate metabolism					
3	Lactate	1.37 (d)	1.37 (d)	1.37 (d)	[CH3]
7	Pyruvate	2.39 (s)	2.31 (s)	2.34 (s)	[CH3]
13	Glucose	–	–	3.20-3.80 (m) 5.15 (d)	[O(CH)OH]
15	Glycolate	3.94 (s)	3.84 (s)	3.87 (s)	[CH2]
Krebs cycle metabolism					
8	Succinate	2.45 (s)	2.41 (s)	2.43 (s)	[CH2]
9	Citrate	2.56-2.62 (d)	2.50-2.60 (d)	2.50-2.60 (d)	[2xCH2]
Purines metabolism					
19	Hypoxanthine	8.19 (s)	8.35 (s)	8.11 (s)	[N(CH)NH]
Hippuric acid					
16	Hippurate	7.50-7.80 (m)	3.87 (d) 7.40-7.80 (m)	7.40-7.60 (m)	[CH]
Carboxylic acids					
20	Formate	8.35 (s)	–	8.47 (s)	[CH]
Alkaloids and derivatives					
21	Trigonelline	8.70-8.80 (m) 9.01 (s)	8.70-8.80 (m) 9.05 (s)	8.60-8.7(m) 9.02 (s)	[NH]

C = control, PD = prediabetes and T2D = type 2 diabetes groups; s = singlet, sb = singlet broad, d = doublet, m = multiplet.

According to findings obtained in the three groups (Figure 1), the highest intensity signal was characteristic of the creatinine region, and those of lowest strength refer to hypoxanthine, formate, and trigonelline. The regions similar to acetate and citrate were more expressive in the T2D group than in other groups. In group C, pyruvate, glycine, glycolate, and urea was more intense. There was a consistent decrease in the urea signal from control to PD to T2D. Notably, the glycine region was not identified in the T2D group, and the characteristic glucose signal was observed only in the T2D group.

### Multivariate analysis

The PCA scores demonstrate that the separation between the groups was not completely visualized for all samples (Figure 2), with evidence of overlap between the C and T2D groups. Thus, it was necessary to use supervised classification techniques.

Different supervised classification techniques were applied to the pre-processed data (Supplementary Table S4), starting with simpler techniques, such as the PCA-LDA, then to more complex techniques, such as the GA-LDA, according to the principle of parsimony [37]. This means that simpler solutions are tested before resorting to a more complex classification method that requires more processing time and greater optimization of parameters [32]. Among the tested algorithms, the GA-LDA was superior to the others, presenting a 100% test set accuracy, sensitivity, and specificity, showing total separation between the samples.

The GA-LDA classification scores are shown in Figure 3. Samples from the training and test sets were discriminated between with 100% accuracy as evidenced by the confusion matrix of the GA-LDA model (Supplementary Table S5), in which it is possible to observe that all training and test samples were classified correctly. Only one sample from the T2D group was classified as a control in leave-one-patient-out cross-validation, reducing the cross-validation accuracy to 96%. Furthermore, the GA-LDA model was additionally validated by Monte-Carlo cross-validation using all the samples (Table 3).

**Table 3.** Confusion matrix and classification rate based on the GA-LDA model with Monte-Carlo cross-validation (1000 iterations, 20% of samples out).

Measured/Predicted groups	C	PD	T2D
C	74.0%	5.1%	20.9%
PD	2.0%	80.5%	17.5%
T2D	14.0%	12.0%	74.0%

C = control, PD = prediabetes, T2D = type 2 diabetes.

In the Monte-Carlo cross-validation, the prediction performance decreased to an average accuracy of 76.2%, where samples from the C and T2D groups were classified 74.0% correctly, while the samples from the PD group were classified 80.5% correctly. 20.9% of samples from the C group and 17.5% from PD were classified as T2D, while 14.0 and 12.0% of samples from T2D were classified as C and PD, respectively. This could show the approximate performance of the GA-LDA classification model with more samples since this validation approach considers 1000 iterations for all investigated samples, with 20% of them left out for validation each time. Furthermore, it shows a tendency to decrease the model accuracy for all classes due to the variability between the samples; nevertheless, it still has prediction accuracies equal to or above 74.0% for all investigated classes.

The list of variables automatically selected by the GA-LDA model is shown in Table 4 and Supplementary Figure S4. These variables represent the ppm values responsible for the perfect classification between the groups. Discrimination of samples in each group was possible.

**Table 4.** List of variables selected by GA-LDA (chemical shifts in ppm), along with the means and standard deviations (SD) for the control (C), prediabetes (PD) and type 2 diabetes (T2D) groups.

ppm	n		PD		T2D		
	Mean	SD	Mean	SD	Mean	SD	p-value
0.69	8.69E-05	1.36E-04	1.10E-04	8.86E-05	7.85E-05	8.17E-05	0.708
0.94	4.82E-04	2.55E-04	3.92E-04	2.53E-04	3.92E-04	3.29E-04	0.685
1.08	1.39E-03	7.69E-04	1.58E-03	9.26E-04	1.18E-03	1.05E-03	0.543
1.22	1.92E-03	1.46E-03	9.82E-04	4.43E-04	1.09E-03	1.15E-03	0.091
1.51	6.89E-04	3.49E-04	6.51E-04	4.60E-04	5.23E-04	5.26E-04	0.617
1.73	1.06E-03	8.88E-04	7.56E-04	4.61E-04	5.03E-04	4.30E-04	0.074
2.94	2.26E-02	4.76E-02	4.41E-02	5.32E-02	6.91E-03	9.01E-03	0.057
3.20	2.84E-03	2.35E-03	2.24E-03	2.94E-03	1.57E-03	1.07E-03	0.322
3.27	7.62E-04	8.77E-04	3.07E-04	3.12E-04	3.85E-03	5.57E-03	0.027*
3.37	1.41E-03	1.15E-03	8.90E-04	5.81E-04	6.35E-03	7.82E-03	0.012*
3.74	5.28E-03	3.84E-03	3.52E-03	2.99E-03	6.46E-03	6.10E-03	0.277
3.75	4.71E-03	2.41E-03	2.84E-03	2.18E-03	9.82E-03	8.67E-03	0.009**
3.99	6.86E-03	1.28E-02	5.16E-03	8.23E-03	3.27E-03	7.77E-03	0.630
4.73	4.92E-02	1.84E-02	4.69E-02	3.96E-02	4.24E-02	2.87E-02	0.841
4.85	2.53E-03	9.59E-04	3.20E-03	2.90E-03	1.72E-03	1.08E-03	0.114
5.50	5.98E-04	7.54E-04	6.57E-04	4.91E-04	2.48E-04	3.24E-04	0.095
5.90	1.19E-03	1.30E-03	6.45E-04	5.46E-04	2.17E-04	3.69E-04	0.012*
6.87	3.71E-05	1.45E-04	2.88E-05	9.64E-05	−1.61E-05	3.25E-05	0.297
7.13	6.14E-05	1.73E-04	2.50E-04	3.35E-04	9.66E-05	2.27E-04	0.167
8.79	−5.79E-07	3.90E-05	−8.21E-07	2.15E-05	4.27E-05	1.51E-04	0.422
8.96	−2.18E-05	1.06E-05	−1.52E-05	9.45E-06	−1.25E-05	9.81E-06	0.069
9.29	−1.74E-05	7.88E-06	−7.77E-06	1.00E-05	−8.92E-06	8.72E-06	0.025*

ANOVA.

* (*p* < 0.05).

** (*p* < 0.01).

Of the 21 variables selected by GA-LDA, five variables (3.27, 3.37, 3.75, 5.90 and 9.29 ppm) showed significant differences, with 3.75 ppm being the most distinct. These regions are considered complex and require continuity in metabolomic analyses to enable chemical characterization.

## Discussion

Our study reinforces the objectivity of ^1^H NMR techniques applied to urine samples to discriminate metabolites characteristic of T2D or PD using the GA-LDA model. We identified twenty-one metabolite regions in the urine of individuals with glycemic alterations. The chemical shifts of metabolites identified in urine from the groups of our study are similar to results obtained from urine analysis using the ^1^H NMR technique in diabetic rats, type 1 diabetic patients, and older adults [38]. Some metabolites such as glycine, urea, glucose, acetate, citrate, and creatinine are highlighted in these findings. Four of these signals showed a more significant difference in the T2D group than the C and PD groups in the 3.27, 3.37, 3.59, 3.75, and 9.29 ppm ranges. The supervised data analysis indicated the GA-LDA model as the algorithm with the best accuracy, sensitivity, and specificity in discriminating group metabolites.

Unlike the other groups, the urine spectra of the T2D group showed high-intensity signals corresponding to the glucose region. Hyperglycemia is an essential indicator for predicting and diagnosing T2D. In urine samples, it is common for the glucose region to show an elevated signal in groups with hyperglycemia or DM [39]. On the other hand, the absence of a glucose peak in the prediabetic group is explained because patients with prediabetes typically do not excrete glucose in the urine, as their blood glucose levels, although elevated, do not usually exceed the renal threshold for glucose reabsorption, which is approximately 180 mg/dL (10 mmol/L) in most individuals [40]. Metabolites such as citrate, acetate, glycine, pyruvate, hippurate, trigonelline, and succinate are involved in glucose homeostasis.

The high peak in the citrate region of the T2D group strengthens the discussion about hyperglycemia associated with the presence of this metabolite [41]. In renal functionality, reabsorption of most molecules occurs through sodium cotransport in the proximal tubule. These effects are partly due to competition generated with other substrates, such as citrate, through excessive filtration of glucose thus preventing its renal reabsorption [42].

In diabetic rats, high concentrations of citrate and acetate were also detected in the urine using the ^1^H NMR technique. The authors suggested that the increase in the concentration of Krebs cycle intermediates, such as citrate, may have occurred due to systemic stress caused by hyperglycemia or local effects on renal tubular transport. The high excretion of acetate, the final product of fatty acid oxidation, can be explained by the increased β-oxidation of fatty acids in the chain of T2D pathophysiological events, causing an increase in acetyl-CoA and, consequently, in acetate production [43].

In our study, it was impossible to identify the region characteristic of glycine in the T2D group, which only occurred in the PD and C groups, with lower intensity in the PD group. Regarding the inclusion of glycine as a new biomarker of PD, it was speculated that a low glycine concentration in urine may be associated with a failure in glucose homeostasis [17,44]. Glycine assumes significant functions in cellular signaling, regulating both central and peripheral glucose homeostasis [40]. Moreover, it acts directly as an agonist of receptors (GlyRs) and serves as a coagonist of N-methyl-D-aspartate. Through its interaction with GlyRs, this amino acid effectively mitigates chronic systemic inflammation and insulin resistance [44]. Furthermore, it has the potential to enhance insulin secretion from pancreatic β cells, albeit with constraints attributed to the inhibitory influence of NMDA receptors on these cells.

The less expressive signs of pyruvate were found in the urine of individuals from the T2D and PD groups. Low pyruvate concentrations were also observed in the ^1^H NMR urine spectra of T2D-induced rats. Since pyruvate is a product of glucose metabolism, the mechanisms involved indicate that the increase in hyperglycemia inhibits glycolytic enzymes (phosphofructokinase, pyruvate kinase, hexokinase) that prevent pyruvate production. This reduction impairs the formation of acetyl-CoA, reduces the activity of the Krebs cycle, and consequently causes mitochondrial dysfunction [39].

We also observed that the characteristic regions of hippurate are more reduced in the PD and T2D groups than in the C group. A recent human study showed that urinary concentrations of hippurate are negatively associated with BMI, body weight, and HOMA-IR. Additionally, this metabolite exhibited a reverse correlation with insulin resistance, steatosis, hypertension, and obesity. A high concentration of this metabolite in urine would be advantageous due to its role in augmenting pancreatic β cells [45].

Trigonelline was visibly low in the three groups, but mainly in the T2D group. Considering that this group showed worse metabolic control, this finding agrees with the observation that over five years in T2D, high urinary concentrations of trigonelline are associated with a decrease in HbA1c1 [17]. Trigonelline has hypoglycemic, neuroprotective, and antitumor effects. The hypoglycemic effect is attributed to its ability to boost insulin secretion and the generation of pancreatic β-cells, alongside a reduction in their apoptosis [17].

In our findings, the T2D and C groups presented a signal with a certain intensity in the region characteristic of succinate. Elevated succinate concentrations in plasma and urine are linked to obesity and T2D [35]. Despite this, these individuals have low succinate receptor (SUCNR1) concentrations, which can stimulate inflammation, glucose tolerance, and metabolic stress. There are also reports of succinate elevation in response to physiological adaptations such as physical exercise, in which this metabolite can regulate energy homeostasis [46]. In our study, it was impossible to test associations between this metabolite and the presence of obesity.

Regarding the significant difference in urea signal intensity, we attribute it to a combination of factors, including variations in renal function, amino acid metabolism, fasting time, and hydration status among participants. NMR studies indicate that the most abundant constituent of urine in healthy individuals is urea, which accounts for 80 to 90% of urinary nitrogen loss [3]. In turn, hyperglycemia is directly associated with changes in renal function. Lower urea and creatinine values were observed in individuals with diabetes, with or without risk of complications, suggesting that urea is an essential biomarker in diabetes-induced metabolic changes [47]. Therefore, the higher urea levels in the control group may be attributed to a better renal capacity of healthy individuals to excrete urea. In addition, this group’s tendency towards a higher protein intake may also explain our findings.

We did not identify notable chemical shifts detected by the GA-LDA due to the complexity of certain signal regions and the lack of necessary information to enable this characterization. Signals of 3.75 and 5.9 ppm may indicate glucose, but they are questioned as metabolic products in humans. Additional analyses are required for clarification, including the utilization of other biologically relevant atomic nucleotides such as 13 C, 15 N, 31 P, 23Na, and 19 F. These nucleotides are instrumental in comprehending the biochemical pathways associated with the metabolism of amino acids, lipids, carbohydrates, and nucleic acids.

The use of the GA-LDA model has been highlighted in metabolomics studies in the health area. A study using Fourier transform infrared spectrometry spectra differentiated blood plasma samples from pregnant women with gestational diabetes from control samples (healthy pregnant women) based on the GA-LDA model. In addition, this algorithm demonstrated 100% accuracy, sensitivity, and specificity when compared with other models [48]. These findings show the GA-LDA model’s usefulness even with other spectroscopic methods. We did not find in the literature that the GA-LDA model was used in studies involving urine analysis by ^1^H NMR in individuals with T2D.

A primary limitation of this study is its sample size; nonetheless, the statistical power to differentiate between the groups was calculated, ranging from 59 to 74%. Larger datasets will increase statistical power, resulting in more robust group separation. Additional limitations include the ^1^H NMR analysis by only one nucleus and the non-identification of important signals for further discussion since there is a lack of disease-specificities. The metabolites in this study are implicated in various diseases and conditions, so they cannot be used solely to signal T2D. Further experimental analysis, such as liquid chromatography and mass-spectrometry, is paramount to filtering these metabolite candidates to those associated with T2D. At the present moment, this preliminary study can distinguish between controls, PD, and T2D groups based solely on the ^1^H NMR data and find possible metabolite candidates, highlighting the innovation of this study in the sense of discriminating urine samples from individuals with glycemic alterations using a robust algorithm (GA-LDA) and thus contributing to research on metabolomics and chronic diseases. In this context, it is emphasized that while ^1^H NMR spectrometers and their recurring expenses are costly, NMR provides valuable information, with the cost of analyzing a single sample being relatively low. Ultimately, the overall cost of analysis is generally reduced. High-flux NMR techniques employing automated liquid handling procedures can detect significant amounts of metabolite information in just a few minutes, thereby reducing labor intensity [8,49].

We also obtained dietary data from the study participants, which will be used to discuss approaches in the nutrimetabolomic field. The relationship between diet and metabolites points to molecular mechanisms involved in the diagnosis and prognosis of diseases [4]. This approach helps with prevention strategies and clinical approaches for T2D.

## Conclusion

The ^1^H NMR technique and the GA-LDA model were demonstrated to be accurate methods for discriminating urine metabolomic profiles in case-control studies involving individuals with PD and T2D. These approaches suggest using urine as an eligible biospecimen, making it a biological resource that, in addition to being non-invasive, is potentially useful for clinical and population studies on metabolomics and T2D.

## Supplementary Material

Supplemental Material

supplementary_tables.docx

## Data Availability

Due to privacy concerns, the raw data will not be fully disclosed. The data that support the findings of this study are available from the corresponding author upon reasonable request.
