# Supplementary material for: Nuclear magnetic resonance combined with genetic algorithm with linear discriminant analysis (GA-LDA) is a suitable model for discriminating urinary metabolomic profiles of individuals with glycemic disorders
Source: Ann Med. 2025 Oct 6;57(1):2566870. doi: 10.1080/07853890.2025.2566870 (PMC12502107; doi:10.1080/07853890.2025.2566870)
Supplement: Supplemental Material [file IANN_A_2566870_SM8135.zip › Suppl_Fig/Figure caption.docx]

**Figure S1.**^1^H NMR spectra of the control (C), prediabetes (PD) and type 2 diabetes (T2D) groups.

**Figure S2**. Expansion of the NMR spectra of the control (C), prediabetes (PD) and type 2 diabetes (T2D) groups in the region of 0 - 5.5 ppm.

**Figure S3.** Expansion of the NMR spectra of the control (C), prediabetes (PD) and type 2 diabetes (T2D) groups in the region of 6.5 - 9.3 ppm.

**Figure S4**. (a) All variables selected by the GA-LDA model responsible for classification between groups; (b) variables selected by GA-LDA for relative intensities below.
