# Supplementary figures and images for: Nuclear magnetic resonance combined with genetic algorithm with linear discriminant analysis (GA-LDA) is a suitable model for discriminating urinary metabolomic profiles of individuals with glycemic disorders
Source: Ann Med. 2025 Oct 6;57(1):2566870. doi: 10.1080/07853890.2025.2566870 (PMC12502107; doi:10.1080/07853890.2025.2566870)

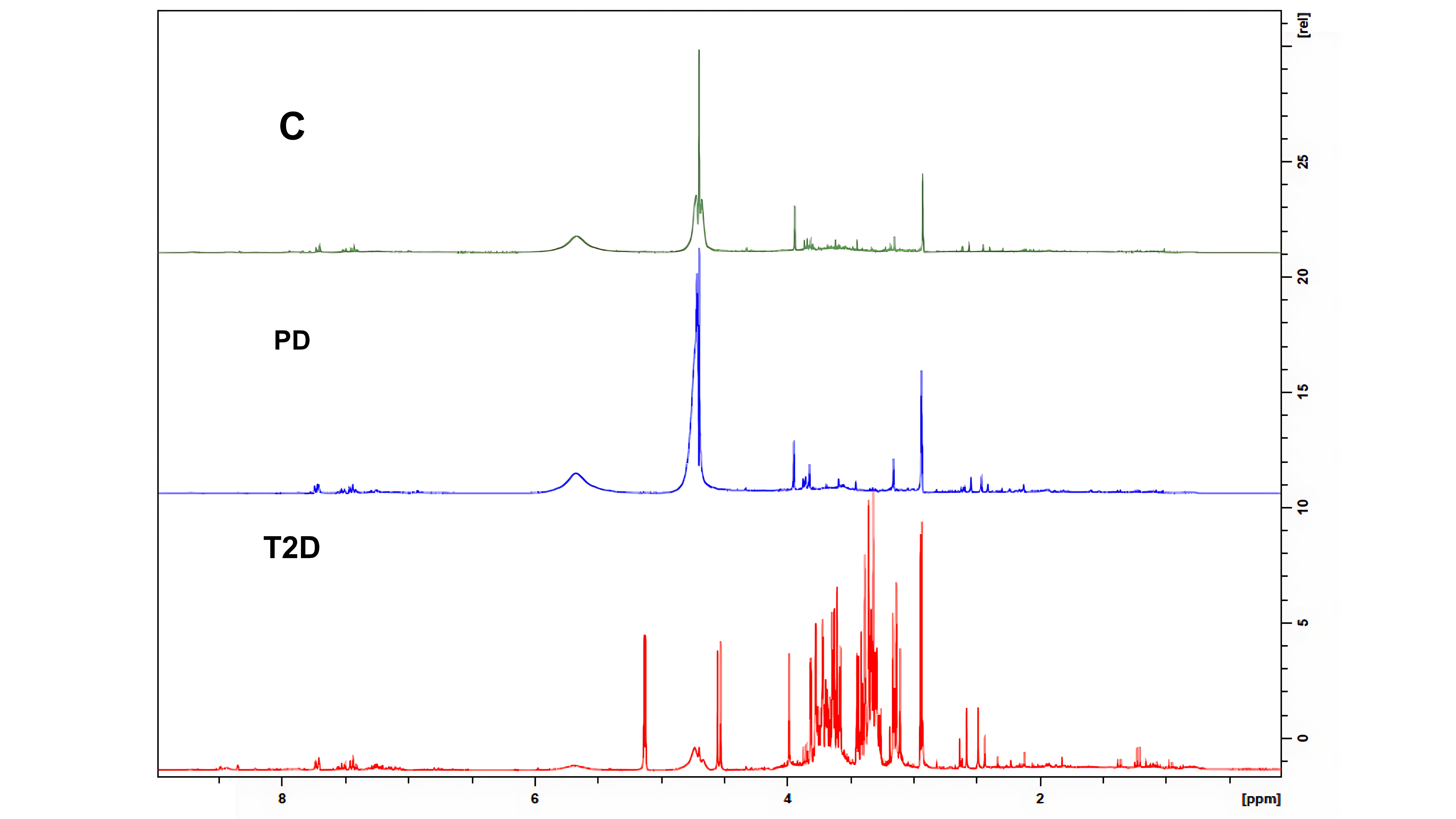

Supplement: Supplemental Material [file IANN_A_2566870_SM8135.zip › Suppl_Fig/Figure S1.png]

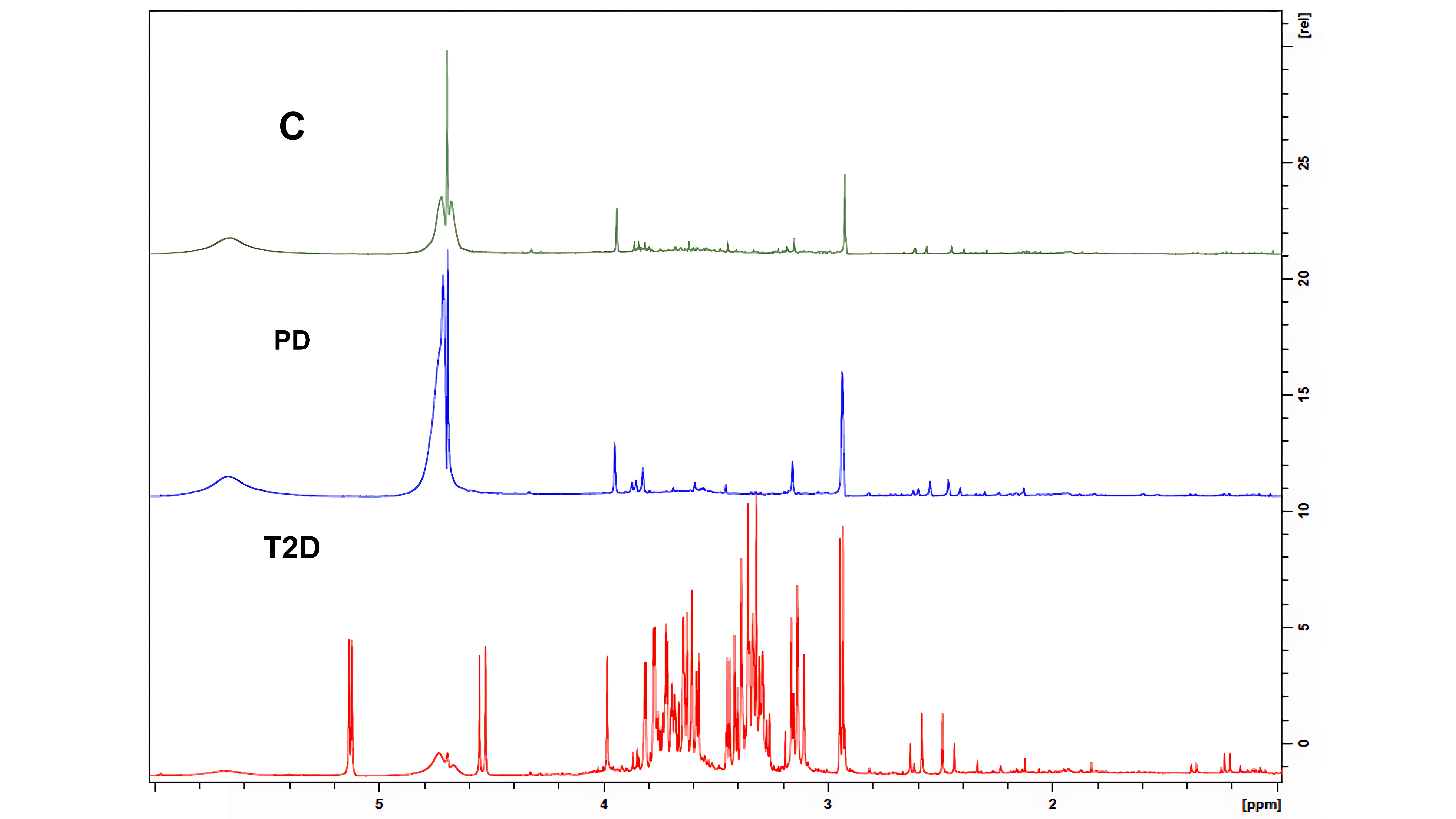

Supplement: Supplemental Material [file IANN_A_2566870_SM8135.zip › Suppl_Fig/Figure S2.png]

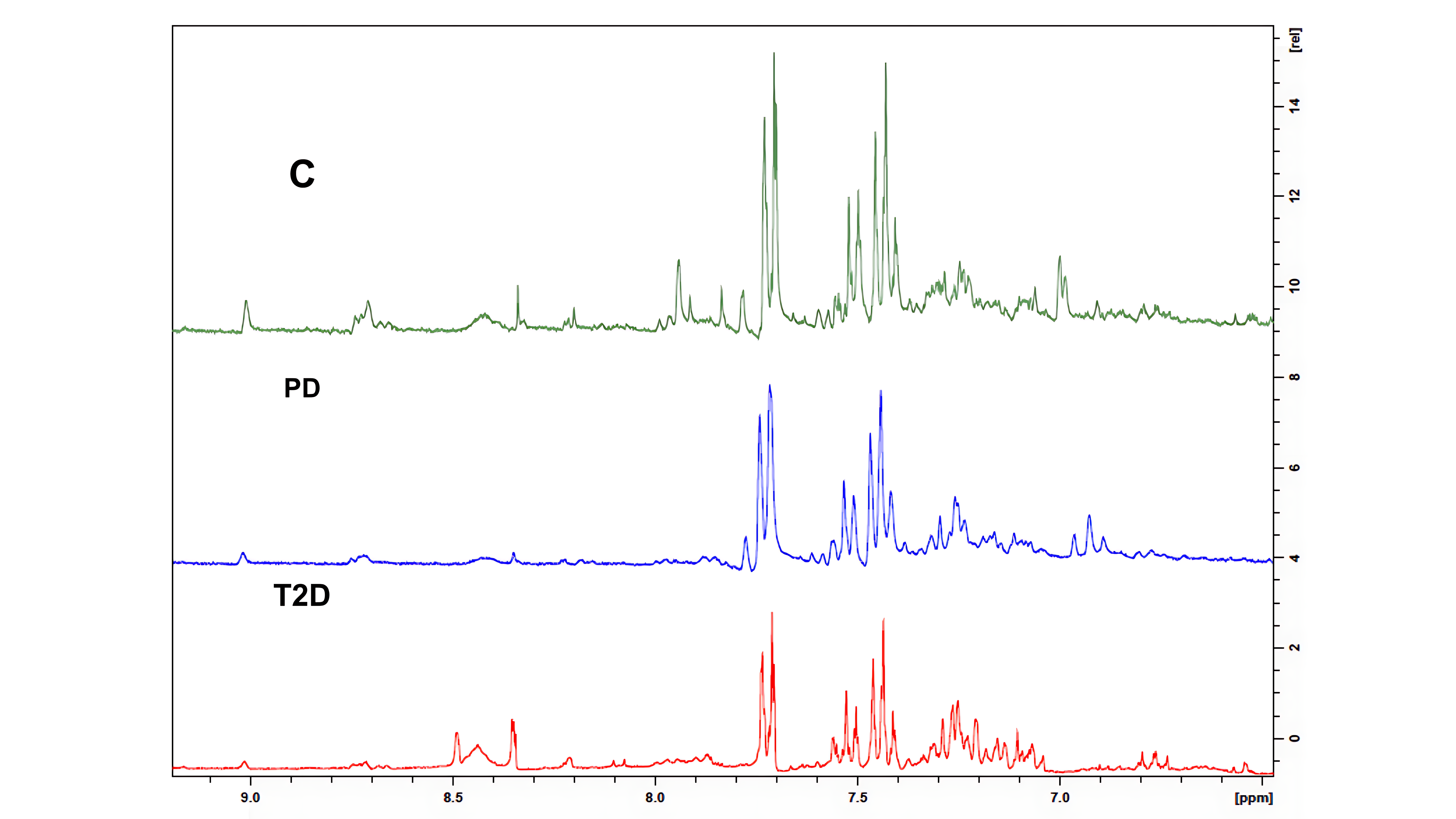

Supplement: Supplemental Material [file IANN_A_2566870_SM8135.zip › Suppl_Fig/Figure S3.png]
