# Supplementary material for: Nuclear magnetic resonance combined with genetic algorithm with linear discriminant analysis (GA-LDA) is a suitable model for discriminating urinary metabolomic profiles of individuals with glycemic disorders
Source: Ann Med. 2025 Oct 6;57(1):2566870. doi: 10.1080/07853890.2025.2566870 (PMC12502107; doi:10.1080/07853890.2025.2566870)
Supplement: supplementary_tables.docx [file IANN_A_2566870_SM3661.docx]

**SUPPLEMENTARY TABLES**

**Nuclear Magnetic Resonance combined with Genetic Algorithm with linear discriminant analysis (GA-LDA) is a suitable model for discriminating urinary metabolomic profiles of individuals with glycemic disorders.**

Papa, AWFS et al.

**Table S1**. Number of samples selected for training and testing of building supervised models

| Set | Number of samples | | | Total |
| --- | --- | --- | --- | --- |
|  | **CONTROL** | **PD** | **T2D** |  |
| Training | 7 | 8 | 12 | 27 |
| Test | 4 | 4 | 4 | 12 |

Control group (C); prediabetes (PD); type 2 diabetes (T2D).

**Table S2**. Distribution of participants according to the diagnostic criteria of glycemic control and lipide profile

| **Variables1,2** | **C** | **PD** | **T2D** | **p-value** |
| --- | --- | --- | --- | --- |
|  | (n=12) | (n=16) | (n=11) |  |
| **Fasting glucose (%)** | |  |  | **0,000** |
| Glycemia <100 | 11 (100)b | 4 (33)a | 3 (19)a |  |
| Glycemia, range 100 - 125 | 0a | 8 (67)b | 2 (13)a |  |
| Glycemia >126 | 0b | 0b | 11 (69)a |  |
| **HbA1c (%)** |  |  |  | **0,000** |
| <5,7 | 11 (100)b | 1 (8)a | 1 (6)a |  |
| ≥5,7 e <6,5 | 0C | 11 (92)b | 6 (38)a |  |
| ≥6,5 | 0b | 0b | 9 (56)a |  |
| **Systolic pressure (%)** |  |  |  | **0,221** |
| SP great | 5 (50) | 1 (11) | 1 (9) |  |
| SP normal | 4 (40) | 4 (44) | 4 (36) |  |
| Pre-hypertension | 0 | 2 (22) | 3 (27) |  |
| SP 1 stage | 1 (10) | 1 (11) | 1 (9) |  |
| SP 2 stage | 0 | 0 | 2 (18) |  |
| SP 3 stage | 0 | 1 (11) | 0 (0%) |  |
| **Total cholesterol (%)** |  |  |  | **0,12** |
| <190 | 4 (36) | 6 (50) | 12 (75) |  |
| ≥190 | 7 (64) | 6 (50) | 4 (25) |  |
| **HDL-c (%)** |  |  |  | **0,01** |
| HDL-c <40 | 0 (0)b | 2 (17)a,b | 8 (50)a |  |
| HDL-c >40 | 11 (100)b | 10 (83)a,b | 8 (50)a |  |
| **Triglycerides (%)** |  |  |  | **0,192** |
| <150 | 9 (82) | 6 (50) | 8 (50) |  |
| >150 | 2 (18) | 6 (50) | 8 (50) |  |
| **LDL (%)** |  |  |  | **0,313** |
| LDL <70 | 1 (9) | 0 | 4 (25) |  |
| LDL <100 | 1 (9) | 4 (33) | 4 (25) |  |
| LDL <130 | 5 (46) | 4 (33) | 6 (38) |  |
| LDL >130 | 4 (36) | 4 (33) | 2 (13) |  |

Control group(C); prediabetes (PD); type 2 diabetes (T2D). Data are presented in percentages in (%). Reference values ​​for the glycemic profile according to the SBD (2019); reference values ​​for blood pressure and lipid profile according to the Brazilian Society of Cardiology (Faludi et al., 2017). Different letters on the same line denote a significant difference between means, (p<0.05) Chi-square test.

**Table S3.** Daily dietary intake of energy, macronutrients, fiber, cholesterol, and sodium of participants

| **Variables** | **C**  (n=11) | **PD**  (n=12) | **T2D**  (n=16) | | **p-value** |
| --- | --- | --- | --- | --- | --- |
| **Energy (kcal)** | 1600 (1333-2569) | 1658 (1590-1808) | | 1574 (1269-1788) | 0,412 |
| **Carbohydrate** |  |  | |  | 0,110 |
| kcal/d | 904 (736-1248) | 848 (796-1232) | | 804 (696-892) |  |
| g/d | 226 (184-312) | 212 (199-308) | | 201 (178-223) |  |
| total de calorias (%) | 57 | 51 | | 51 |  |
| **Protein** |  |  | |  | 0,734 |
| kcal/d | 316 (256-524) | 300 (292-372) | | 296 (240-392) |  |
| g/d | 79 (64-131) | 75 (73-93) | | 74 (60-98) |  |
| total calories (%) | 20 | 18 | | 19 |  |
| **Total fat** |  |  | |  | 0,316 |
| kcal/d | 378 (333-765) | 432 (405-486) | | 369 (333-486) |  |
| g/d | 42 (37-85) | 48 (45-54) | | 41 (37-54) |  |
| total calories (%) | 24 | 26 | | 24 |  |
| **Saturated fat** |  |  | |  | 0,737 |
| kcal/d | 135 (126-279) | 153 (126-180) | | 153 (99-198) |  |
| g/d | 15 (14-31) | 17 (14-20) | | 17 (11-22) |  |
| total fat calories (%) | 36 | 35 | | 41 |  |
| **Monounsaturated fat** |  |  | |  | 0,848 |
| kcal/d | 117 (108-252) | 126 (117-135) | | 126 (81-162) |  |
| g/d | 13 (12-28) | 14 (13-15) | | 14 (9-18) |  |
| total fat calories (%) | 31 | 29 | | 34 |  |
| **Polyunsaturated fat** |  |  | |  | 0,610 |
| kcal/d | 63 (63-126) | 72 (63-81) | | 72 (45-81) |  |
| g/d | 7 (7-14) | 8 (7-9) | | 8 (5-10) |  |
| total fat calories (%) | 17 | 17 | | 20 |  |
| **Total fiber (g)** | 15 (15-24) | 18 (15-21) | | 17 (12-26) | 0,999 |
| **Cholesterol (mg)** | 420 (419-379) | 302 (202-463) | | 432 (251-536) | 0,355 |
| **Sodium (mg)** | 1014 (604-3286) | 1951 (1204-2367) | | 1524 (1075-1773) | 0,543 |

Control group (C); prediabetes (PD); type 2 diabetes (T2D). Recommendations for carbohydrate, 45 to 60%, protein, de 15 to 20% and total fat, 20 to 35% of the total energy value of the diet (TEV). Fiber: minimum 14 g/1.000 kcal; 20 g/1.000 kcal para T2D (SBD, 2019). Data presented as median (interquartile range). Kruskall-Wallis test; (p<0,05).

**Table S4**. Confusion matrix for the GA-LDA model and the number of samples classified for each group: control (C), prediabetes (PD) and type 2 diabetes (T2D)

|  | Training | | | Cross-validation | | | Test | | |
| --- | --- | --- | --- | --- | --- | --- | --- | --- | --- |
|  | **C** | **PD** | **T2D** | **C** | **PD** | **T2D** | **C** | **PD** | **T2D** |
| C | 7 | 0 | 0 | 7 | 0 | 0 | 4 | 0 | 0 |
| PD | 0 | 8 | 0 | 0 | 8 | 0 | 0 | 4 | 0 |
| T2D | 0 | 0 | 12 | 1 | 0 | 11 | 0 | 0 | 4 |

Control group (C); prediabetes (PD); type 2 diabetes (T2D).

**Table S5.** Supervised classification models tested on a pre-processed data set

| **Algorithm** | **Set** | **Accuracy (%)** | **Sensitivity (%)** | | | **Specificity (%)** | | |
| --- | --- | --- | --- | --- | --- | --- | --- | --- |
|  |  |  | **C** | **PD** | **T2D** | **C** | **PD** | **T2D** |
| PCA-LDA  (10 PCs) | Training | 81 | 70 | 88 | 85 | 90 | 83 | 100 |
|  | CV | 48 | 30 | 38 | 69 | 76 | 78 | 67 |
|  | Test | 57 | 20 | 75 | 80 | 78 | 90 | 67 |
| PCA-QDA | Training | 81 | 80 | 100 | 69 | 90 | 91 | 89 |
|  | CV | 58 | 60 | 63 | 54 | 81 | 87 | 67 |
|  | Test | 79 | 80 | 100 | 60 | 78 | 100 | 89 |
| PLS-DA  (1 LV) | Training | 71 | 70 | 63 | 77 | 71 | 91 | 94 |
|  | CV | 55 | 70 | 25 | 62 | 67 | 91 | 72 |
|  | Test | 64 | 100 | 25 | 60 | 56 | 100 | 89 |
| SVM  (RBF kernel, c = 100, g = 0.01) | Training | 57 | 70 | 0 | 100 | 76 | 100 | 67 |
|  | CV | 44 | 30 | 25 | 77 | 76 | 83 | 61 |
|  | Test | 40 | 20 | 0 | 100 | 89 | 100 | 22 |
| GA-LDA  (22 variáveis selecionadas) | Training | 100 | 100 | 100 | 100 | 100 | 100 | 100 |
|  | CV | 96 | 100 | 100 | 92 | 95 | 100 | 100 |
|  | Test | 100 | 100 | 100 | 100 | 100 | 100 | 100 |

PCA-LDA, principal component analysis with linear discriminant analysis; PCA-QDA, principal component analysis with quadratic discriminant analysis; PLS-DA, partial least squares with discriminant analysis; SVM, support vector machines; GA-LDA, genetic algorithm with linear discriminant analysis; CV, leave-one-patient out cross-validation; control group (C); prediabetes (PD) and, type 2 diabetes (T2D). Parameters in parentheses after the algorithm name indicate the parameters used to build the models.
